# MitoLSDB: A Comprehensive Resource to Study Genotype to Phenotype Correlations in Human Mitochondrial DNA Variations

**DOI:** 10.1371/journal.pone.0060066

**Published:** 2013-04-09

**Authors:** Shamnamole K, Saakshi Jalali, Vinod Scaria, Anshu Bhardwaj

**Affiliations:** 1 Department of Bioinformatics, Stella Maris College, University of Madras, Chennai, Tamil Nadu, India; 2 Genomics and Molecular Medicine, CSIR-Institute of Genomics and Integrative Biology, Delhi, India; 3 Open Source Drug Discovery Unit, Council of Scientific and Industrial Research (CSIR), Delhi, India; University of Utah, United States of America

## Abstract

Human mitochondrial DNA (mtDNA) encodes a set of 37 genes which are essential structural and functional components of the electron transport chain. Variations in these genes have been implicated in a broad spectrum of diseases and are extensively reported in literature and various databases. In this study, we describe MitoLSDB, an integrated platform to catalogue disease association studies on mtDNA (http://mitolsdb.igib.res.in). The main goal of MitoLSDB is to provide a central platform for direct submissions of novel variants that can be curated by the Mitochondrial Research Community. MitoLSDB provides access to standardized and annotated data from literature and databases encompassing information from 5231 individuals, 675 populations and 27 phenotypes. This platform is developed using the Leiden Open (source) Variation Database (LOVD) software. MitoLSDB houses information on all 37 genes in each population amounting to 132397 variants, 5147 unique variants. For each variant its genomic location as per the Revised Cambridge Reference Sequence, codon and amino acid change for variations in protein-coding regions, frequency, disease/phenotype, population, reference and remarks are also listed. MitoLSDB curators have also reported errors documented in literature which includes 94 phantom mutations, 10 NUMTs, six documentation errors and one artefactual recombination. MitoLSDB is the largest repository of mtDNA variants systematically standardized and presented using the LOVD platform. We believe that this is a good starting resource to curate mtDNA variants and will facilitate direct submissions enhancing data coverage, annotation in context of pathogenesis and quality control by ensuring non-redundancy in reporting novel disease associated variants.

## Introduction

Mitochondria are the essential energy-generating organelles in eukaryotes possessing the oxidative phosphorylation system (OXPHOS). Mitochondrial disorders are caused by mutations in mitochondrial genes encoded by nuclear or mitochondrial DNA (mtDNA) [1]. The OXPHOS comprises of five protein complexes and majority of their protein subunits are nuclear encoded with only a subset of 37 genes encoded by mtDNA [2]. Of these 37 genes, 13 are protein subunits, 22 tRNAs, and 2 rRNAs. These genes are essential components of electron transport chain complexes I, III and IV and complex V (ATP synthase) [3]. Mutations in these genes have been associated with a broad spectrum of diseases [4] which are reported as per coordinates of the revised Cambridge reference sequence (NC_012920). At least 1 in every 200 births is thought to have a potentially pathogenic mitochondrial DNA mutation [5]. The disease phenotypes attributed to mutations in mtDNA have diverse and overlapping symptoms and also multi-organ involvement [6]. Many deleterious point mutations have been identified to date, the most frequent ones being the m.3243A>G MELAS mutation [7], the LHON primary mutations [8], and the m.8344A>G MERRF mutation [9]. Others are found less often, while still others have been described only as case studies or in families. The investigation of pathogenic mtDNA mutations has revealed a complex relation between patient genotype and phenotype [10]. The phenotypic variability is due to the peculiarities of mitochondrial properties, such as heteroplasmy, different mutation rates in different tissues and highly polymorphic nature [11]–[13]. Therefore, the patho-mechanisms of mtDNA point mutations are still not very well understood. Furthermore, there appears to be a class of slightly deleterious mutations that modify the risks of developing certain complex diseases or traits [14]. Besides, heteroplasmic and homoplasmic mtDNA have also been observed along with large number of basal polymorphisms in the mitochondrial genome across databases like OMIM [http://www.ncbi.nlm.nih.gov/omim] [15], MitoMap [16], Mitovariome [17]and mtDB [18]. These facts highlight the challenges in assessing the role of mtDNA variants in diseases or phenotypes.

Recent reports indicate role of mitochondrial dysfunction in the pathogenesis of or influence the risk of diseases such as Alzheimer, Parkinson’s, cardiovascular disease including cardiomyopathy, etc. [19]–[21]. But the genotype-phenotype relationship is unclear and debatable [22], [23]. More than 5000 complete or coding-region sequences of publicly available mtDNA were analyzed to study the diversity of the global human population [24]. This study has generated useful data in the form of all possible transitions and transversions and their analysis lead to interesting observations that may help in understanding the role of mtDNA variants in disease. Besides, there has been an increase in DNA variant data resulting from new automatic sequencing technologies [25]. Thus, it is imperative to catalogue this information on a standard web-based platform for sharing and evaluating the potential pathological effects of mtDNA variants. To this end, we have used the Leiden Open (source) Variation Database (LOVD) Software [26], [27] for creating a catalog of human mtDNA variants, through manual curation of data from literature and from public databases. LOVD is a commonly used tool for organizing locus-centric variation data. As of now, MitoLSDB has patient and variant information from 5231 individuals from 675 different populations [24], [28] from 27 different groups including patients with Alzheimer’s disease, Asthanozoospermic, Atypical psychosis, Breast cancer, Diabetes, Angiopathy, Deafness, Glioma, Parkinson’s disease, Teratozoospermic, Thyroid cancer, etc and can be accessed at http://mitolsdb.igib.res.in. MitoLSDB is a Locus-Specific DataBase (LSDB) for human mtDNA genes and provides access to standardized and annotated data compiled from different resources which are otherwise difficult to search and comprehend. This is in line with the objectives of LSDBs, which are expected to contain comprehensive information from disparate resources and are open for direct submissions. It has also been observed that a large amount of variant data from case studies or reports never get published and LSDBs have served as a viable platform for the scientific community to benefit from and actively contribute to [29]. For each variant curated in MitoLSDB, its genomic location as per the Revised Cambridge Reference Sequence, codon and amino acid change for variations in protein-coding regions, frequency, disease/phenotype, population, reference and remarks are listed. MitoLSDB curators have also reported errors documented in literature which includes 94 phantom mutations, 10 NUMTs (Nuclear mitochondrial DNA sequences), six documentation errors and one artefactual recombination. We believe that this is a good starting resource to curate mtDNA variants and will facilitate direct submissions enhancing data coverage, annotation in context of pathogenesis and quality control by ensuring non-redundancy in reporting novel disease associated variants.

## Methods

### Data Collection

The variant data and other patient information of 5139 individuals from different populations were obtained from the study by Pereira et al [24] and the public databases www.phylotree.org [30], accessed between December 2010 and March 2011, and Ian Logan’s website http://www.ianlogan.co.uk/checker/genbank.htm [31], which belongs to 26 different groups and a set of 92 complete genomes from sporadic ataxia patients [28]. A set of PERL scripts were developed to extract variant data from the sources. The dataset obtained from Pereira et al’s study [24] gives the details on sample ID, variant, reference, haplotype reported, origin/ethnicity and phenotype. However, this dataset only provides variant positions in each sample. This information was complemented with variant detail with help of other resources. These variants are reported as per the coordinates of the revised rCRS (Revised Cambridge Reference Sequence) [GenBank: NC_012920.1 gi: 251831106]. We have also reported errors documented in literature which includes 94 phantom mutations [31], 10 NUMTs [32], 6 documentation errors and one artefactual recombination [31] in the remarks section of the database. This data is converted to match the ‘import file’ specifications of LOVD.

### Data Integration into Database

The database is customized on the LOVD platform which is supported on the backend by a MySQL relational database management system. Links are provided to genes to assist the user in searching detailed information related to the gene. In addition, plug-ins have been created to export the data to a standard meta-tagged format for interoperability with other resources. This would aid the user to have a genome centered and holistic view of the variants and this would be helpful in interpreting the biological impact of variations. In human mtDNA there are five instances of overlapping bases among genes and thus these have been mapped to both genes. Codon assignments for mtDNA are different from the universal genetic code and thus the alternate codon table is utilized for reporting codon changes [33]. The database provides for each variant, information on its genomic location, gene name, frequency, phenotype, tissue, sample information, methodology, codon and amino acid change for protein variation, variant submission link, advance search options and registration guidelines for a new submitter.

### Metadata with the Standards of LOVD & MeSH Terms

Controlled vocabulary for reporting phenotype obtained from MeSH has been used to ensure standardization in reporting disease phenotypes [Supplementary Table 1]. Also, MitoLSDB has all obligatory and recommended metadata listed as minimal requirements for LSDBs [http://www.gen2phen.org/post/lsdb-minimal-requirements-d34-compared-lovd-and-lsdb-xml-format, accessed in March 2012] [Supplementary Table 2].

## Results and Discussion

**Figure 1:**
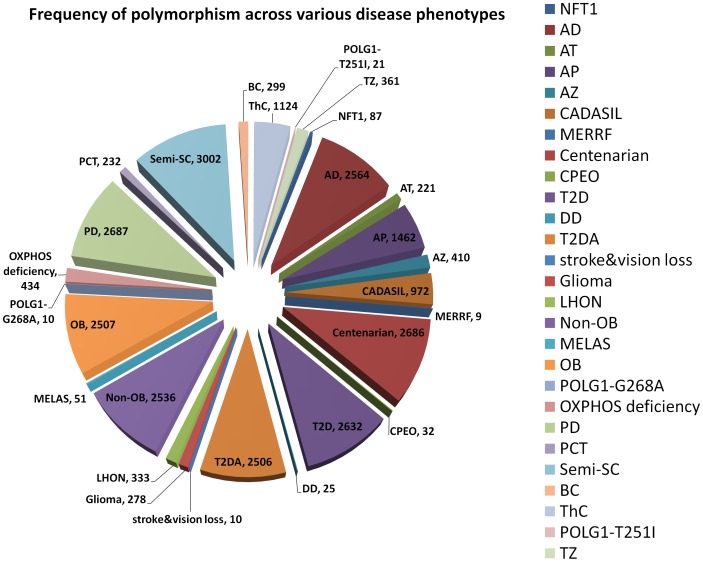
Frequency distribution of variants across various disease phenotypes. The abbreviations in the pie are : AD - Alzheimer’s disease; AT – Ataxia; AP - Atypical Psychosis; OB – Obese; Non-OB - Non obese; PCT - primary cancerous tissue; BC - breast cancer; ThC- thyroid cancer; TZ – Teratozoospermic; semi-SC - semi-supercentenarian; T2D - Type 2 diabetes; T2DA - Type 2 Diabetes with Angiopathy; DD - Diabetes and deafness; NFTT1 - Neurofibromatosis type I; AZ –Asthanozoospermic; PD - Parkinson’s disease; POLG1-T251I - genotype: POLG1 variant T251I; POLG1-G268A - genotype: POLG1 G268A; MELAS – Mitochondrial Encephalopathy, Lactic acidosis and Stroke like episodes; LHON – Leber’s Hereditary Optic Neuropathy; MERRF – Myoclonic Epilepsy with Raged Red Fibers; CADASIL – Cerebral Autosomal Dominant Arteriopathy with Subcortical Infarcts and- Leukoencephalopathy; Stroke& vision loss - stroke-like episodes, lactic acidosis, exercise intolerance similar to that seen in MELAS; two episodes of transient central vision loss similar to that seen in LHON; CPEO – chronic progressive external opthalmoplegia; OXPHOS deficiency; Glioma; Centenarian.

MitoLSDB comprise of variant data from 5231 individuals from 675 populations which belong to 27 different categories [Figure 1 & Supplementary Table 1]. The base changes are reported as per the genomic coordinates of rCRS so that this data can be compared easily with other datasets. Overall, 132397 variants are catalogued in MitoLSDB. Of these 5147 are unique genomic variants, wherein 4226 belong to protein-coding genes, 538 to rRNA genes and 383 are tRNA variants. Of the 4226 protein-coding variants, 158 are nucleotide ambiguities. In the remaining 4068 protein coding variants, 1349 and 2719 are non-synonymous and synonymous changes, respectively with 1066, 528 and 2474 at first, second and third codon positions, in that order. Presence of variation at specific codon position may also be related to the strength of association with the phenotype. In disease association studies, variations occurring with high frequency in patients as compared to normal individuals are considered to be disease associated. For most of the disease phenotypes included in MitoLSDB, there is no information on the normal population variants and hence it is not possible to report disease association based on frequency differences. We have instead reported the frequency of each variant within each population that may assist in evaluating the pathogenic status of these variants during subsequent data analyses.

**Figure 2:**
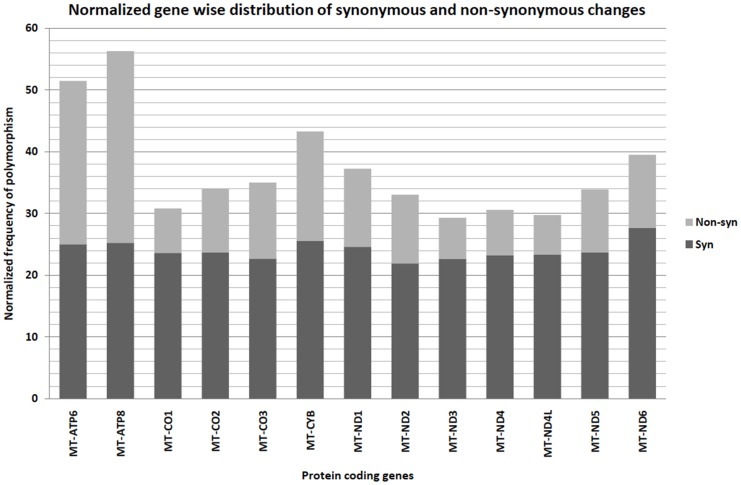
Normalized gene-wise distribution of synonymous & non-synonymous changes. The number of polymorphic sites in each protein coding gene is divided by the gene length to obtain normalized values of polymorphism across these genes in mtDNA. The graph shows the distribution of synonymous and non-synonymous changes in each gene.

A closer look at the data highlights that MT-ATP8 shows the maximum number of non-synonymous variations after normalization for gene length. Similarly, MT-ND6 shows the maximum number of synonymous changes [Figure 2]. However, MT-ND2 and MT-ND4L harbor least number of synonymous changes and non-synonymous changes, respectively.

**Figure 3:**
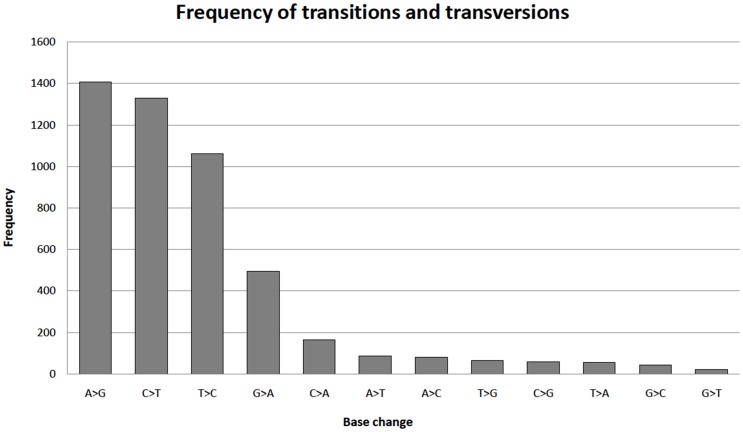
Frequency of transitions and transversions. This graph shows the frequency of base changes in the entire dataset. The frequency of transitions (4293) is higher than transversions (575).

MT-CYB shows the maximum number of polymorphisms with frequency one, which is 2048 in number. For example, the variant m.15326A>G is seen in all the 77 samples from Finland CADASIL population and there are many more variants captured with frequency one. m.8860A>G, m.750A>G, m.15326A>G are some of the variants seen repeatedly in different samples from various populations. This highlights the systemic involvement of these mitochondrial variants in diseases or phenotypes. The statistics of base changes shows a clear skew towards transitions (4293) being more common as compared to transversions (575) [Figure 3]. The A>G transitions are most common, while G>T transversions occurs with least frequency. As stated earlier, variations are more frequency at the third position in codons as compared to second position.

In addition to customizing mtDNA variation and patient data in LOVD format, MitoLSDB also follows the metadata standards for reporting locus specific datasets. In addition to reporting the phenotype as published by authors, MeSH terms have been used to report the same. Standard data formats and ontologies ensure better understanding of terms and reusability of data across platforms. As the standards evolve over time with enhanced understanding of genotype-phenotype correlations, it is imperative to curate data systematically to ensure seamless adaptation to improved metadata structure, if possible, in automated fashion.

### Errors Detected and Reported

We have reported a number of errors documented in literature for the datasets integrated in MitoLSDB. These errors include 94 phantom mutations, 10 NUMT (Nuclear mitochondrial DNA sequences) contaminations, 6 documentation errors, and one sequence with artefactual recombination. Data reported by Pereira et al [24] are directly retrieved from GenBank. It has been observed that many mtDNA sequences available in GenBank are reported errors and unintended mistakes [31], [34]. Many of these errors have already been reported in literature or sometimes even corrected by the authors. Unfortunately in several instances the new corrected versions of sequences have not been updated in GenBank [31].These documentation errors include missing variants, phantom mutations and artefactual recombinants that may lead to wrong conclusions. Missing variants are those that are expected in a particular mtDNA haplotype according to its haplogroup status. For example, the sequence [GenBank:DQ826448] lacks an additional nine expected variants to group that sequence into haplogroup M7b1. Phantom mutations are defined by the exclusive presence of a rare transversion [31]. These are systematic artefacts generated in the course of the sequencing process. The amount of artefacts depends not only on the automated sequencer and sequencing chemistry employed, but also on other lab-specific factors [35] and it is also observed that the pattern of phantom mutations differs significantly from that of natural mutations [36]. In particular, phantom mutation hotspots could lead to spurious mapping of somatic mutations and to misinterpretations in clinical mtDNA studies [1]. Another type of error reported in GenBank mtDNA sequences is the NUMTs. These are the mitochondrial DNA sequences in the nuclear genome [37] (nuclear mitochondrial pseudogenes) which on accidental amplification can pose a serious problem for mitochondrial disease studies [32]. Primers designed for amplification of mtDNA can potentially anneal with sequences in nuclear genome that present at high homology to mtDNA. In fact NUMTs have already been mistaken as heteroplasmic positions in the case of reported association of mutations in MT-CO1 and MT-CO2 with development of Alzheimer’s disease, which were later shown to be an artefact resulting from the accidental amplification of nuclear mitochondrial pseudo genes [38]. Studies on artefactual recombinations [39], [40] and various missing mutations [41], [42] have also been used to report the status of variants in the data sets used in MitoLSDB. For example, the mtDNA sequence [GenBank:DQ834258] may be a recombinant since it bears m.8701A>G (MT-ATP6) and m.9540T>C (MT-CO3), characteristic of non-N status; but this sequence was misclassified as haplogroup HV, due to artefactual recombination [31]. This sequence is reported as ‘recombinant sequence’ in the database remarks section.

### Conclusion

**Figure 4:**
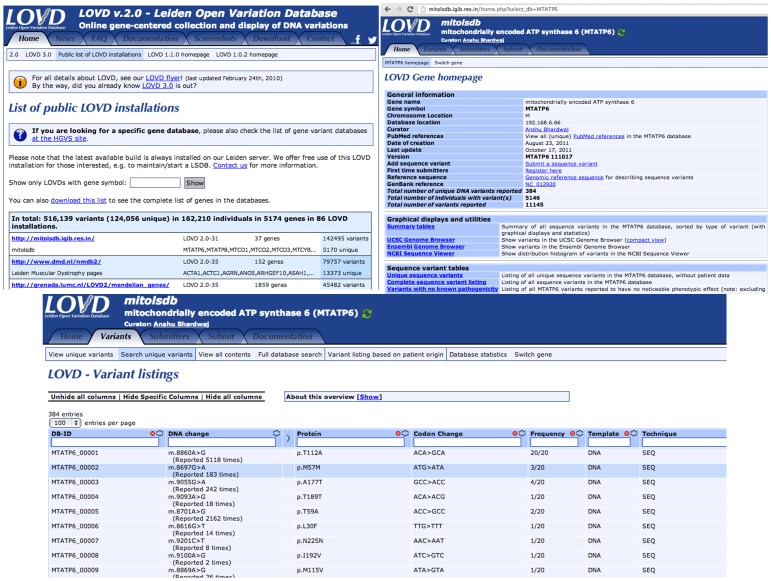
Screenshot of the MitoLSDB database. Screenshot shows the MitoLSDB home page for a gene and the variant listing. It also shows that MitoLSDB is listed as a public LOVD installation.

MitoLSDB is a systematic compilation of mtDNA variant information and is expected to facilitate the submission of novel variants by the users. This is proposed as a starting resource to curate mitochondrial DNA variants, which would facilitate researchers in genotype-phenotype studies and also streamline the task of reporting novel mutations. It would also allow cross-comparison of different mtDNA association studies and help understand the molecular correlates of mitochondrial disease phenotypes, which otherwise is a very daunting and challenging task given the complexity of mitochondrial genetics.Variants are integrated in MitoLSDB in a standard updatable format, with a very user friendly interface [Figure 4]. We believe that MitoLSDB may work as a central repository for reporting novel pathogenic variants and provide a solution to documented issues in context of spurious reports and faulty conclusions on disease association status of mtDNA variants [43].

MitoLSDB is a freely accessible website that allows researchers to retrieve mitochondrial genome variation data on 5231 individuals from various populations. Unlike other available sources, users can browse and obtain the variation data gene wise. It also allows the user to list the variants based on patient origin. Contrasting to other existing resources MitoLSDB provides data on variants caused by insertions and deletions. The MitoLSDB curators do not report the missing mutation or haplogroup information in the first version of the database because of the ambiguities reported in the haplogroup status, which may lead the researcher to wrong conclusions. We have reported the available corrected errors in the database remarks column. It would be the best to get these ambiguities confirmed by the original authors.

### Future Perspectives

To the best of our knowledge, MitoLSDB is the largest repository of mtDNA variants systematically standardized and presented using the LOVD platform. The curators have integrated data from 675 populations comprising of 5231 individuals and 5147 unique variants. We are attempting to make the data interoperable with various genomic databases and computational workflows, which would facilitate easy and automated analysis of the variants. This would facilitate researchers in genotype-phenotype studies. MitoLSDB would also allow cross-comparison or meta-analysis of different mtDNA association studies and help understand the molecular correlates of mitochondrial disease phenotypes, which otherwise is a very daunting and challenging task given the complexity of mitochondrial genetics. It has been demonstrated earlier that publications contain a significant number of reporting errors that have been corrected or reported by curators and submitters of LSDBs [29]. We expect a similar trend for mtDNA variations and believe that community participation will further enhance data coverage, improved annotations in context of pathogenic status of variants and quality checks for spurious reports and correctness of the submitted data.

## Supporting Information

Table S1 **The table lists the phenotype as obtained from literature along with their ethnic background.** The third column list the number of individuals in each phenotype category. The last column provides MeSH terms for the phenotype as obtained from http://www.ncbi.nlm.nih.gov/mesh. The controlled vocabulary is used to ensure standardization in reporting disease phenotypes.
(PDF)

Table S2 **The table lists the metadata used in MitoLSDB in column one and the status of corresponding metadata listed as minimal requirements for LSDBs in the second column.**
(PDF)
