# Correction: MitoLSDB: A Comprehensive Resource to Study Genotype to Phenotype Correlations in Human Mitochondrial DNA Variations

**DOI:** 10.1371/journal.pone.0236810

**Published:** 2020-07-23

**Authors:** Shamnamole K, Saakshi Jalali, Vinod Scaria, Anshu Bhardwaj

The URL listed in this article [1] for MitoLSDB is no longer functional. Since the article's publication, MitoLSDB has been migrated to https://ab-openlab.csir.res.in/mitolsdb/home.php.

The corresponding author’s current affiliation has changed. Anshu Bharwaj is now affiliated with CSIR Institute of Microbial technology, Chandiagarh, India. Their updated email address is: anshu@imtech.res.in

## References

[1] K S, Jalali S, Scaria V, Bhardwaj A (2013) MitoLSDB: A Comprehensive Resource to Study Genotype to Phenotype Correlations in Human Mitochondrial DNA Variations. PLoS ONE 8(4): e60066 10.1371/journal.pone.0060066 23585830 PMC3621970

