# Supplementary material for: MitoLSDB: A Comprehensive Resource to Study Genotype to Phenotype Correlations in Human Mitochondrial DNA Variations
Source: PLoS One. 2013 Apr 9;8(4):e60066. doi: 10.1371/journal.pone.0060066 (PMC3621970; doi:10.1371/journal.pone.0060066)
Supplement: Table S1 — The table lists the phenotype as obtained from literature along with their ethnic background. The third column list the number of individuals in each phenotype category. The last column provides MeSH terms for the phenotype as obtained from http://www.ncbi.nlm.nih.gov/mesh. The controlled vocabulary is used to ensure standardization in reporting disease phenotypes. (PDF) [file pone.0060066.s001.pdf]

Phenotype	Ethnicity/Population	Number of individuals	MeSH Heading
Alzheimer disease	Chiba Japan	96	Alzheimer disease
Asthenozoospermic	Portugal	20	Sperm Motility
Atypical psychosis	Japan	57	Psychotic Disorders
Breast cancer	Italy	19	Breast Neoplasm
	Romania	1	
CADASIL	Finland	77	CADASIL
Centenarian	Gifu Japan	11	
	Gifu Japan	11	Aged, 80 and over
	Tokyo Japan	85	
chronic progressive external opthalmoplegia (CPEO)	Bonn, Germany	1	Ophthalmoplegia, Chronic Progressive External
diabetes and deafness	The Netherlands	1	Diabetes and deafness, maternally inherited
Diabetes Type II	Aichi Japan	96	Diabetes Mellitus, Type 2
	Ashkenazi Jew; Belarus	1	
Diabetic with angiopathy	Tokyo Japan	96	Diabetic Angiopathies
genotype: POLG1 G268A	Russia: Belgorod	1	DNA-Directed DNA Polymerase
genotype: POLG1 variant T251I	Russia: Belgorod	1	DNA-Directed DNA Polymerase
Glioma	Italy	16	Glioma
LHON	Central China	2	Optic Atrophy, Hereditary, Leber
	China	1	
	Italy	7	
	Brazil	1	
	Tuvan, Russia, North-East Siberia	1	
	slavic, Siberia, Russia	2	
MELAS	Turkey	1	MELAS Syndrome
	Vietnam	1	
	Germany	1	
MERRF	Germany	1	MERRF Syndrome
neurofibromatosis type 1		4	Neurofibromatosis 1
Non-obese young male	Aichi Japan	96	No suitable term
Obese young male	Aichi Japan	96	No suitable term
OXPHOS deficiency	The Netherlands: Nijmegen	28	Mitochondrial Diseases
Parkinson disease	Tokyo; Japan	96	Parkinson Disease
primary cancerous tissue	China	10	No suitable term
Semi-supercentenarian	Japan	112	Aged, 80 and over
stroke-like episodes, lactic acidosis, exercise intolerance similar to that seen in MELAS; two episodes of transient central vision loss	Ashkenazi, Jew, Poland	1	
Teratozoospermic	Portugal	23	No suitable term
Thyroid cancer	Italy	64	Thyroid Neoplasms
	Albania	1	
	Chile	1	
