# Supplementary material for: MitoLSDB: A Comprehensive Resource to Study Genotype to Phenotype Correlations in Human Mitochondrial DNA Variations
Source: PLoS One. 2013 Apr 9;8(4):e60066. doi: 10.1371/journal.pone.0060066 (PMC3621970; doi:10.1371/journal.pone.0060066)
Supplement: Table S2 — The table lists the metadata used in MitoLSDB in column one and the status of corresponding metadata listed as minimal requirements for LSDBs in the second column. (PDF) [file pone.0060066.s002.pdf]

**Metadata**	**Status in LOVD**
Variant/Exon	Recommended
Variant/DNA_genomic	Obligatory
Variant/DNA_coding	Recommended
Variant/RNA	Obligatory
Variant/Protein	Obligatory
Variant/DBID	Obligatory
Variant/Reference	Obligatory
Variant/DNA_published	Recommended
Variant/Detection/Template	Obligatory
Variant/Detection/Technique	Obligatory
Variant/DNA_remark	Recommended
Variant/Frequency	Recommended
Variant/Origin	Recommended
Variant/Allele	Recommended
Variant/Pathogenicity	Recommended
Patient/Patient_ID	Obligatory
Patient/Phenotype/Disease	Obligatory
Patient/Remarks	Recommended
Patient/Origin/Geographic	Recommended
Patient/Origin/Ethnic	Recommended
Patient/Gender	Recommended
ID_submitterid	Obligatory
Variant/HGNC gene Symbol	Obligatory
